# Proteomic analysis of bladder biopsies from interstitial cystitis/bladder pain syndrome patients with and without Hunner’s lesions reveals differences in expression of inflammatory and structural proteins

**DOI:** 10.1186/s12894-020-00751-x

**Published:** 2020-11-07

**Authors:** Elijah P. Ward, Sarah N. Bartolone, Michael B. Chancellor, Kenneth M. Peters, Laura E. Lamb

**Affiliations:** 1 Department of Urology, Beaumont Health System, 3811 W. 13 Mile Road, Suite 504, Royal Oak, MI 48073 USA; 2 Oakland University William Beaumont School of Medicine, Rochester, MI USA

**Keywords:** Interstitial cystitis, Proteomics, HPLC, Urinary bladder

## Abstract

**Background:**

Interstitial cystitis/bladder pain syndrome is a bladder disease usually characterized by pain, urgency, and frequency. Interstitial cystitis is currently classified into two subtypes, with and without Hunner’s lesions. However, the underlying etiology of interstitial cystitis and its subtypes are largely unknown.

**Methods:**

To better understand the biological changes in the bladder of interstitial cystitis/bladder pain syndrome patients, we directly analyzed bladder tissue of interstitial cystitis patients, both those with Hunner’s lesions and those without. Proteins in the bladder biopsies were analyzed using nanoscale high-performance liquid chromatography-tandem mass spectrometry. Disease subgroups were compared and significantly expressed proteins were mapped using STRING to determine protein associations and functions.

**Results:**

We found that patients with Hunner’s lesions had significant increases in inflammatory and endoplasmic reticulum stress proteins, with a decrease in cellular adhesive proteins, compared to patients without Hunner’s lesions. These patients also exhibited a decrease in proteins associated with the Rap1 signaling pathway, which regulates cell proliferation and wound healing. When comparing diseased and non-disease-apparent tissue in patients with Hunner’s lesions, diseased tissue exhibited a decrease in ubiquitination proteins.

**Conclusions:**

In summary, there are significant differences in protein expression found in the bladders of interstitial cystitis patients with and without Hunner’s lesions, indicating a disturbance in proteins associated with cellular adhesion, proliferation, protein processing, and wound healing.

## Background

Interstitial cystitis (IC) is a disease of the bladder that can be characterized by urinary frequency, urgency, pelvic pressure, and pain in the absence of a urinary tract infection. The projected incidence of IC varies, ranging from 45 per 100,000 to as many as 307 per 100,000 individuals [1, 2]. However, the incidence may be higher than estimated as IC is commonly misdiagnosed. The pathogenicity of IC is currently unknown, though multiple theories have been proposed [3]. There are two IC/BPS subtypes, the first is IC/BPS with Hunner’s lesions (HIC; also known as Hunner’s ulcers), representing 5–10% of IC/BPS patients [4]. HIC is diagnosed via cystoscopy and is characterized by areas of increased inflammation and cellular degradation. HIC patients often have more severe symptoms, including increased pain, sensitivity, and changes in bladder permeability [5]. The second subtype is IC/BPS without Hunner’s lesions (NHIC). NHIC is difficult to diagnose given the lack of clear physiological changes; patients may or may not have areas of submucosal glomerulations, or small areas of submucosal bleeding in the bladder wall distinct from Hunner’s lesions. As a result, diagnosis is mainly based on exclusion of other conditions with similar symptoms [6].

While IC/BPS is a widely studied urological condition, the molecular differences between healthy patients, NHIC and HIC remain poorly understood. Several studies have illustrated that IC bladders, compared to healthy bladders, display decreased cellular proliferation and cell permeability [7, 8]. Additionally, biomarker studies have identified distinct differences between HIC and NHIC bladders; urine from HIC patients have higher concentrations of inflammatory cytokines and urothelial breakdown products [9]. The differences between diseased and non-disease-apparent (NDA) areas within one bladder also remains unknown. Therefore, the goal of our study is to use nanoscale high performance liquid chromatography coupled to tandem mass spectrometry (nHPLC-MS/MS) on bladder biopsies of diseased and NDA tissue to identify distinct protein differences between NHIC and HIC. This method is highly sensitive, which is beneficial when working with limited sample volume, such as bladder biopsies. This is the first time nHPLC-MS/MS has been used to examine IC bladders directly. Using this innovative approach, we found that HIC patients exhibit increased inflammatory proteins and decreased cellular adhesion proteins in the urothelium, as well as increased ER stress, suggesting a possible difference in disease etiology between HIC and NHIC.

## Methods

### Sample collection and storage

This study was approved by the institutional IRB (IRB# 2009-218) and written consent for all participants was obtained. Disease state was determined during cystoscopy by a trained urologist. Bladder samples were obtained from 4 female patients with HIC and 4 female patients with NHIC during routine medical cystoscopy. Two biopsies were collected from each patient: one from a diseased site (glomerulations in NHIC patients, Hunner’s lesions in HIC patients), and another from an adjacent non-disease-apparent (NDA) site as determined by an experienced urologist. 5 mm × 5 mm samples of bladder tissue were snap-frozen and stored at − 80 °C. Patients were asked to complete the validated Interstitial Cystitis Symptom Index and Problem Index (ICSI/ICPI) to measure lower urinary tract symptoms (LUTS) and impact on patient quality of life (QOL) [10]. ICSI scores range from 0 to 19 and ICPI scores range from 0 to 16, with higher scores representing more severe symptoms and impact [3].

### Sample processing

Deidentified samples were sent to MSBioworks for processing and analysis. Samples were blinded and randomized to the researchers. Samples were washed in 1 × PBS and mechanically disrupted in a Bullet Blender (NextAdvance) with 1.6 mm stainless steel beads in 100µL of modified RIPA lysis buffer (2% SDS, 150 mM NaCl, 50 mM Tris pH 8, 1X Roche complete protease inhibitor). Samples were incubated at 60 °C for 30 min, then clarified by centrifugation. The protein concentration of each extract was determined by Qubit fluorometry (Invitrogen). 10 μg of each sample was processed by SDS-PAGE using a 10% Bis–Tris NuPage mini-gel (Invitrogen) in the MES buffer system. The migration window (2 cm lane) for each sample was excised into ten equally sized bands, each was processed by in-gel digestion with trypsin using a ProGest robot (DigiLab). Samples were first washed with 25 mM ammonium bicarbonate, followed by acetonitrile. Samples were reduced with 10 mM dithiothreitol at 60 °C, followed by alkylation with 50 mM iodoacetamide at room temperature. Digestion was performed with trypsin at 37 °C for 4 h. Finally, samples were quenched with formic acid and the supernatant was analyzed via nHPLC-MS/MS.

### Liquid chromatography and mass spectrometry

Sample supernatants were analyzed by nHPLC-MS/MS with a NanoAcquity HPLC system (Waters) linked to a Q Exactive mass spectrometer (ThermoFisher). Digested proteins were loaded on a Luna C18 resin (Phenomenex) trapping column and eluted over a Luna C18 resin 75 µm analytical column at a flow rate of 350 nL/min. The mass spectrometer was operated in data-dependent mode, with the Orbitrap operating at 70,000 FWHM for MS and 17,500 FWHM for MS/MS. The fifteen most abundant ions were selected for MS/MS.

### Data processing and statistical analysis

Data was analyzed using Mascot with the following parameters: the database used was SwissProt Human (concatenated forward and reverse plus common contaminants), with the enzyme set to Trypsin/P. Fixed modifications were set to Carbamidomethyl (C), while variable modifications included Acetyl (N-term), Deamidation (N,Q), Oxidation (M), and Pyro-Glu (N-term Q). Mass values were monoisotopic. Peptide and Fragment Mass Tolerances were 10 ppm and 0.02 Da, respectively. 2 max missed cleavages were allowed. After Mascot processing, data was parsed into Scaffold for validation and filtering. Data were filtered using a 1% protein and peptide false discovery rate (FDR) while requiring at least 2 unique peptides per protein. To determine approximate relative abundance of proteins within a given sample and between samples, normalized spectral abundance factor (NSAF) was calculated based on the following equation:

$${\text{NSAF}} = \left( {{\text{SpC}}/{\text{MW}}} \right)/\Sigma \left( {{\text{SpC}}/{\text{MW}}} \right)_{{\text{N}}}$$

where SpC = Spectral Counts, MW = Protein Molecular Weight (in kDa), and N = Total Number of Proteins. Significant proteins were determined by comparing average NSAF values for diseased and adjacent NDA tissues to generate fold change values, as well as a T-test for significance. Proteins were considered significant if they had a fold change difference ≥ 4 and a *p* value ≤ 0.05. Interactions and relationships between these proteins were mapped using STRING. Proteins were connected by function and interaction, with interactions predicted by experimental data, database searches, text mining, and co-expression.

### STRING mapping

Significant proteins were uploaded to STRING [11] (string-db.org) and mapped to predict protein–protein interactions, represented by colored lines between protein nodes. These relationships include known interactions determined by experiments (purple) and database mining (teal), predicted interactions via gene neighborhood (green), co-occurrence (blue), or gene fusion (red), or from text mining (yellow), co-expression (black), or protein homology (light blue).

## Results

**Fig. 1:**
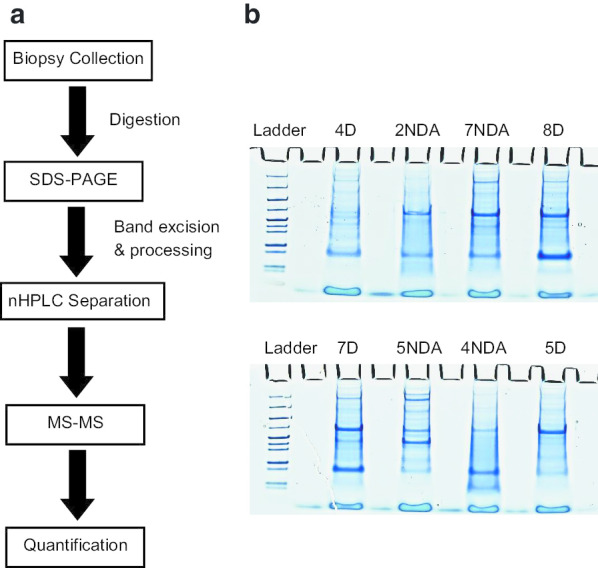
Experimental workflow of IC bladder biopsies. **a** Patient biopsies were digested and separated by SDS-PAGE before analysis via nHPLC-MS/MS. Data was searched using Mascot, and a list of detected proteins was created for each sample. Significant proteins were mapped for associations and shared function via STRING. **b** 10 μg of digested bladder tissue was loaded onto an SDS-PAGE and separated. Bands were excised and digested prior to nHPLC-MS/MS. Samples were blinded and randomized to avoid bias. Patient number corresponds to patient number in Table 1. D = Disease tissue biopsy; NDA = Non-disease apparent tissue biopsy

Our group was interested in understanding the differences in protein expression between bladder biopsies from HIC and NHIC patients These biopsies were processed and analyzed via nHPLC-MS/MS (Fig. 1), resulting in 2,625 unique proteins identified between all patient samples. Patients also completed the ICSI and ICPI questionnaires, with HIC patients reporting higher average scores than NHIC patients (Table 1). This is consistent with what has been previously reported. When comparing protein of interest between subgroups, we focused on proteins that showed a fold change of ≥ 4 × and a significant difference between groups (e.g. *p* ≤ 0.05).

**Table 1:** Patient demographics

Patient #	Age	Gender	IC subtype	ICSI score	ICPI score
1	60	F	HIC	19	16
2	79	F	HIC	15	16
3	71	F	HIC	N/A	N/A
4	55	F	HIC	18	15
5	36	F	NHIC	10	9
6	63	F	NHIC	19	16
7	43	F	NHIC	11	13
8	53	F	NHIC	15	12

### Diseased areas in HIC patients exhibit decreased expression of ubiquitination proteins compared to NDA areas in HIC patients

First, we compared diseased areas of HIC tissue to NDA areas of HIC bladders to determine protein differences. 18 proteins were found to be significantly altered when comparing NDA to diseased HIC tissues. Three of these proteins were linked to ubiquitination (Table 2).

**Table 2:** Diseased Hunner’s IC biopsies compared to non-disease-apparent Hunner’s IC biopsies

Accession ID	Gene name	Description	Fold change	*p* value (*p* ≤ 0.05)
		Ubiquitination (down-regulated) *3 of 18 significant proteins*	NDA HIC/diseased HIC	
P68036	*UBE2L3*	Ubiquitin-conjugating enzyme E2 L3	7.565	0.0119
P63279	*UBE2I*	SUMO-conjugating enzyme UBC9	7.288	0.0347
Q7Z6Z7	*HUWE1*	E3 ubiquitin-protein ligase	6.997	0.0040

### Diseased areas in NHIC patients show minimal changes in protein composition when compared to NDA areas

Comparing diseased areas of NHIC bladder tissue to NDA areas revealed 13 proteins were significant. These proteins function in various stages of peptide and lipid metabolism, as well as partial function in cytoskeleton organization.

### Areas of disease in HIC patients show increased expression of inflammatory proteins and decreased expression of cellular adhesion proteins compared to areas of disease in NHIC patients

**Fig. 2:**
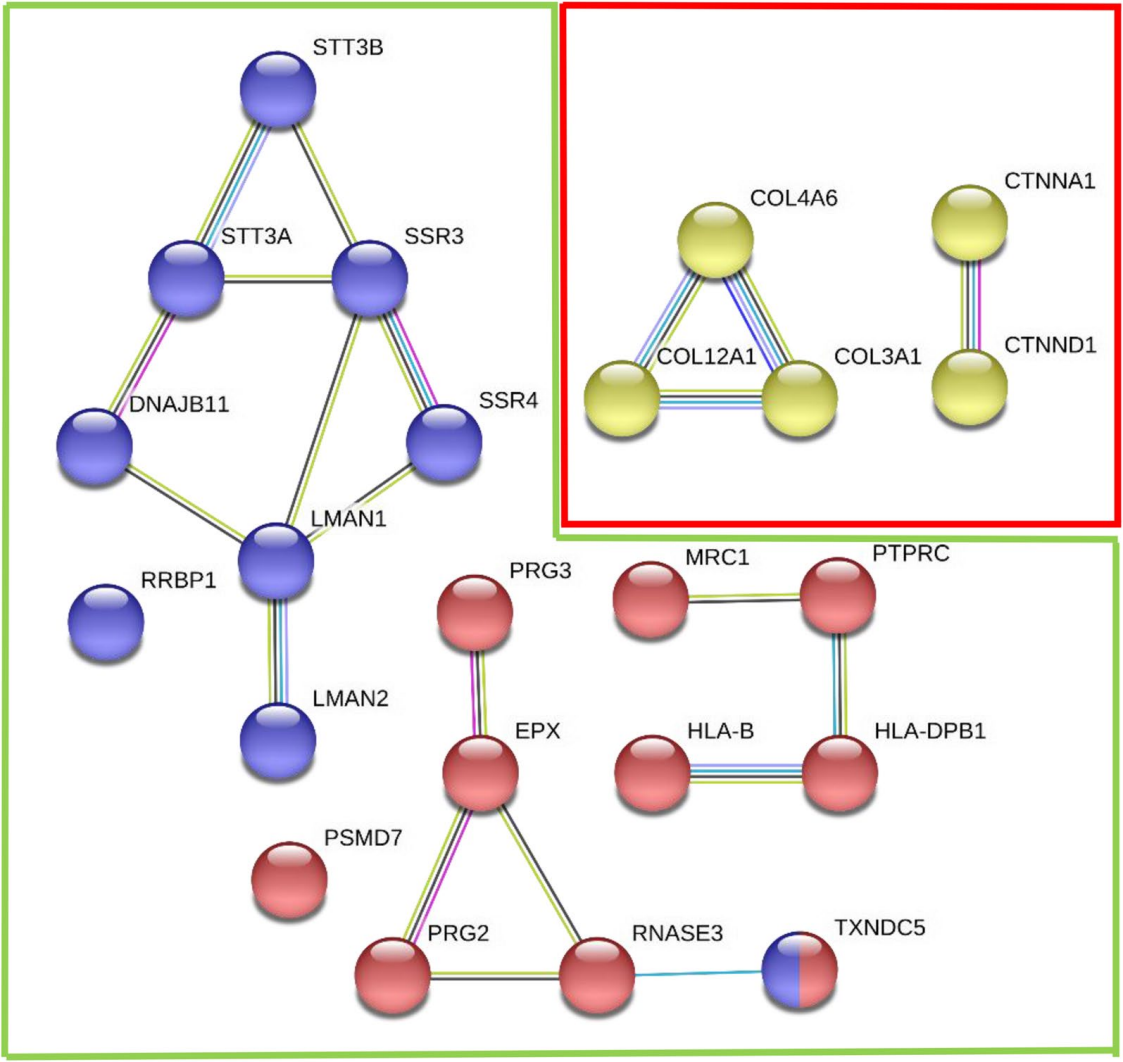
Diseased HIC samples exhibited increased expression of immune response and ER stress proteins, with a decrease in cellular adhesion proteins. Association map for proteins differentially expressed in diseased Hunner’s IC biopsies compared to diseased non-Hunner’s IC biopsies. Nodes in the green box represent proteins with increased expression in diseased HIC tissue, while nodes within the red outline represent proteins with decreased expression. Red nodes represent proteins with an immune response function, blue represents ER protein processing/stress response, and yellow represent cellular adhesive proteins. Lines between nodes indicate reported associations as detailed in “Methods” section

Next, we compared diseased areas of HIC patients with diseased areas of NHIC patients. 34 proteins were found to be significantly increased when comparing diseased HIC tissue to diseased NHIC tissue. STRING’s protein mapping (Fig. 2) revealed that ten of these proteins are involved in immune response and are associated with inflammatory diseases, such as asthma and type I diabetes mellitus, while nine of these proteins are associated with protein processing in the endoplasmic reticulum (ER) (Table 3). In addition, five proteins involved in collagen formation and cellular adhesion were significantly decreased in HIC patients compared to NHIC patients (Table 3).

**Table 3:** Diseased Hunner’s IC biopsies compared to diseased non-Hunner’s IC biopsies

Accession ID	Gene Name	Description	Fold change (FC ≥ 4)	*p* value (*p* ≤ 0.05)
		Immune and inflammatory response (up-regulated) *10 of 34 significant proteins*	Diseased HIC/diseased NHIC	
P11678	*EPX*	Eosinophil peroxidase	74.7	0.0184
P08575	*PTPRC*	Tyrosine phosphatase C	45.9	0.0163
P12724	*RNASE3*	Eosinophil cationic protein	19.6	0.0072
P13727	*PRG2*	Bone marrow proteoglycan precursor	13.6	0.0046
P01889	*HLA-B*	HLA class I histocompatibility antigen, B-41	10.5	0.0011
P51665	*PSMD7*	26S proteasome non-ATPase regulatory subunit	9.16	0.0481
P04440	*HLA-DPB1*	HLA class II histocompatibility antigen, DP beta 1 chain	6.77	0.0079
Q8NBS9	*TXNDC5*	Thioredoxin domain-containing protein 5	5.55	0.0065
P22897	*MRC1*	Mannose-receptor C-type 1	5.91	0.0303
Q9Y2Y8	*PRG3*	Proteoglycan 3	4.46	0.0038
		ER protein processing & ER stress (up-regulated) *9 of 34 significant proteins*		
Q8TCJ2	*STT3B*	Dolichyl-diphosphooligosaccharide-protein glycosyltransferase subunit STT3B	22.2	0.026
Q9UNL2	*SSR3*	Translocon-associated protein subunit gamma	13.6	0.0135
Q2P2E9	*RRBP1*	Ribisome-binding protein 1	11.2	0.0119
Q9UBS4	*DNAJB11*	DnaJ Homolong subfamily B member 11	11.1	0.0008
P46977	*STT3A*	Dolichyl-diphosphooligosaccharide-protein glycosyltransferase subunit STT3A	8.79	0.0248
P49257	*LMAN1*	Protein ERGIC-53	5.97	0.0244
Q8NBS9	*TXDNC5*	Thioredoxin domain-containing protein 5	5.55	0.0065
Q12907	*LMAN2*	Vesicular integral-membrane protein VIP36	5.27	0.0028
P51571	*SSR4*	Translocon-associated protein subunit delta	4.04	0.0091
		Cellular adhesion (down-regulated) *5 of 42 significant proteins*	Diseased NHIC/Diseased HIC	
Q14031	*COL4A6*	Collagen alpha-6(IV)	12.6	0.0144
P35221	*CTNNA1*	Catenin alpha-1	9.54	0.0005
O60716	*CTNND1*	Catenin delta-1	5.69	0.0288
P02461	*COL3A1*	Collagen alpha-1(III)	4.62	0.0039
Q99715	*COL12A1*	Collagen alpha-1(XII)	4.46	8.13e-5

### NDA areas in HIC patients exhibit decreased expression of protein metabolism and Rap1 signaling proteins compared to NDA areas in NHIC patients

**Fig. 3:**
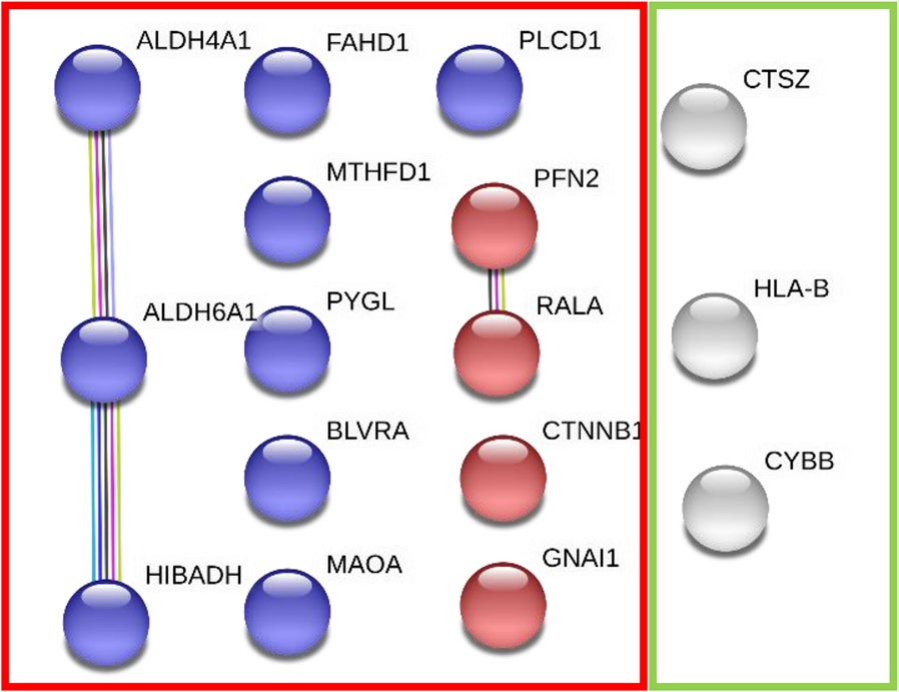
NDA HIC tissues exhibited increased expression of immune response proteins, with decreased protein expression for Rap1 signaling and general metabolism. Association map for proteins differentially expressed in NDA Hunner’s IC biopsies compared to NDA non-Hunner’s IC biopsies. Nodes in the green box were increased in NDA HIC samples, while nodes in the red box were decreased. White nodes represent immune response proteins, while blue and red nodes represent protein metabolism and Rap1 signaling proteins, respectively. Lines between nodes indicate reported associations as detailed in “Methods” section

Finally, we sought to compare NDA tissues of HIC patients with NDA tissue of NHIC patients. 50 proteins were found to be significantly altered when comparing NDA NHIC tissue to NDA HIC tissue. STRING’s protein mapping (Fig. 3) revealed nine proteins involved in protein metabolism and four proteins linked to the Rap1 signaling pathway, which is involved in cell proliferation and adhesion, as well as three proteins involved in the immune response (Table 4).

**Table 4:** Non-disease-apparent Hunner’s IC biopsies compared to non-disease-apparent Non-Hunner’s IC biopsies

Accession ID	Gene name	Description	Fold change (FC ≥ 4)	*p* value (*p* ≤ 0.05)
		Immune and inflammatory response (up-regulated) *3 of 11 significant proteins*	NDA HIC/NDA NHIC	
P30479	*HLA-B*	Human leukocyte antigen B	15.6	0.0039
P04839	*CYBB*	Cytochromeb-245 beta chain	8.11	0.0136
Q9UBR2	*CTSZ*	Cathepsin Z	7.88	0.0037
		Protein metabolism (down-regulated) *9 of 50 significant proteins*	NDA NHIC/NDA HIC	
P30038	*ALDH4A1*	Delta-1-pyrroline-5-carboxylate dehydrogenase	17.9	0.0252
P06737	*PYGL*	Glycogen phosphorylase	8.13	0.0316
P31937	*HIBADH*	3-Hydroxyisobutyrate dehydrogenase	7.73	0.0073
P53004	*BLVRA*	Biliverdin reductase A	5.57	0.0316
P51178	*PLCD1*	1-Phosphatidylinositol 4,5-bisphosphate phosphodiesterase delta 1	5.18	0.0093
Q02252	*ALDH6A1*	Methylmalonate-semialdehyde dehydrogenase	4.66	0.0265
Q6P587	*FAHD1*	Acylpyruvase FAHD1	4.61	0.0235
P21397	*MAOA*	Amine oxidase [Flavin-containing] A	4.61	0.0117
P11586	*MTHFD1*	C-1-tetrahydrofolate synthase	4.28	0.0107
		Rap1 Signaling (down-regulated) *4 of 50 significant proteins*	NDA NHIC/NDA HIC	
P11233	*RALA*	Ras-related protein Ral-A	7.11	0.0026
P35080	*PFN2*	Profilin-2	6.49	0.0124
P63096	*GNAI1*	Guanine nucleotide-binding protein G(i) subunit alpha-1	4.23	0.0266
P35222	*CTNNB1*	Catenin beta-1	4.04	0.0118

## Discussion

To better understand the underlying mechanisms of disease in IC, we sought to determine differences in protein expression between patients with HIC and NHIC. To accomplish this, we collected tissue biopsies from diseased and non-disease apparent areas (NDA) of the bladder from four female patients with HIC and four female patients with NHIC. The tissue samples were processed, and nHPLC-MS/MS was used to analyze the protein compositions. We found significant differences in protein expression in bladder biopsies between patients with HIC and NHIC. Diseased HIC tissues showed a decrease in proteins involved in ubiquitination compared to NDA HIC tissues. Diseased NHIC compared to NDA NHIC tissues showed few differences, mainly in lipid and peptide metabolism. Diseased HIC patient tissues, when compared to diseased NHIC tissues, showed an increase in proteins involved with ER stress and inflammatory response, with a decrease in cell adhesion proteins. Finally, NDA HIC patient tissues also showed a decrease in immune cell adhesion proteins compared to NDA NHIC tissues.

Within the same patient and bladder, the diseased HIC tissue samples had decreased expression of ubiquitination pathway proteins compared to NDA HIC tissue samples. Ubiquitination is a posttranslational modification and plays an important role in regulation of multiple protein pathways, including membrane trafficking and protein turnover involving the ER [12]. Ubiquitination is also important for wound healing and angiogenesis, as well as protein metabolism [13]. This data suggests that the diseased areas of HIC bladders are impaired in their ability to identify and label misfolded or damaged proteins for intracellular proteolysis by the proteasome, which may result in the accumulation of damaged proteins, abnormal protein aggregation, and/or inappropriate association with other proteins. This could subsequently influence oxidative and ER stress, inflammation, and apoptosis, as is common for other chronic conditions, especially neurodegenerative disorders, including Alzheimer’s, Parkinson’s and Huntington’s diseases [14].

Comparing diseased and NDA NHIC biopsies revealed 13 significant proteins. These proteins function primarily in peptide (ADE2) and lipid (APOC-III) metabolism, as well as cytoskeletal formation and organization (Spectrin alpha chain). These results imply that there is not as extreme a difference between areas in the same bladder of NHIC patients, compared to differences seen in HIC patients. There is also no overlap in significant protein expression between the two comparison groups, which could imply a difference in disease etiology.

Comparing areas of disease in HIC patients to NHIC patients, there was a significant increase in proteins associated with inflammation in HIC. The upregulation of inflammatory proteins may indicate an increased immune response within Hunner’s lesions. These proteins have various functions in the mediation of immune processes, including Eosinophil Peroxidase and Tyrosine-Phosphatase C, which had a fold change of 74.7 and 45.9, respectively. These proteins are indicative of increased eosinophil and leukocyte activity. Other immune cell proteins increased in HIC tissue are known to be expressed by monocytes. This increased expression of immune cells is consistent with prior research, as the literature suggests that HIC may have an immune response component [15, 16].

Diseased HIC bladder tissue also showed an increase in proteins involved with ER stress when compared to diseased NHIC tissues. ER stress is a cellular response to improperly folded proteins or high cellular secretory load and may lead to cell death. An increase in the concentration of ER stress proteins in the biopsies of HIC patients may suggest impaired protein processing, which can lead to inflammation and apoptosis. A study in rats with protamine/lipopolysaccharide-induced IC noted an increase in ER stress biomarkers post-procedure, suggesting a link between IC and ER stress [13]. Diseased HIC tissues also showed decreased expression of cellular adhesive proteins, including multiple collagen (collagen alpha-1, collagen alpha-6) and catenin (catenin alpha-1, catenin delta-1) subtypes. These results could imply a weakening of the urothelial lining of the bladder and loss of the barrier function, exposing underlying cell layers to irritants and possibly explaining in part the pain symptoms of patients. Increased ER stress proteins and decreased cellular adhesion proteins in HIC bladders further suggest that the diseased areas of HIC patients are more disrupted and compromised than the diseased areas of NHIC, and also why focal treatment of these areas in HIC can lead to at least temporary symptom relief.

NDA bladder biopsies of HIC patients showed decreased expression of proteins involved in peptide metabolism and protein processing when compared to NDA biopsies from NHIC patients. These patients also showed a decrease in proteins related to Rap1 signaling. The Rap1 signaling pathway controls cell proliferation, cell-to-cell adhesion, and cellular junction formation. The decreased expression of these proteins suggests a weakened cell-to-cell connection in the urothelium of HIC patients and possibly an impaired wound healing response, consistent with the chronic course of IC.

The limitations of this study include the limited number of patient samples, the exclusion of male samples, and the lack of healthy controls. The small number of patient samples reflects the difficultly of obtaining human bladder biopsy samples, which requires an invasive procedure and has some risks including infection and pain. The majority of IC patients are females, therefore we focused on female samples to eliminate gender as a variable. There were several advantages of this study. While other groups have analyzed protein concentrations in bladder tissue from IC patients [17], this is the first time nHPLC-MS/MS has been used for proteomic analysis of IC bladder tissues; nHPLC-MS/MS uses significantly less material compared to normal HPLC, which is important considering how precious these bladder biopsy samples are [18]. This study also directly examined human bladder tissue samples of IC patients, rather than examining human urine or experimental animal models of interstitial cystitis.

## Conclusions

Interstitial cystitis is a disease with unknown etiology. We sought to understand the differences in protein expression in the bladder tissue of patients with two IC subtypes, Hunner’s IC and Non-Hunner's IC. To do this, we collected biopsies from four female HIC patients and four female NHIC patients, processed the biopsies, and analyzed them using nHPLC-MS/MS. In the same bladder, diseased HIC tissues showed a decrease in ubiquitination proteins compared to NDA tissues. When comparing between IC subtypes, HIC patients showed significantly increased inflammatory response and ER stress proteins, with a decrease in cellular adhesive proteins and proteins associated with the Rap1 signaling pathway, which regulates cellular adhesion and wound healing. These results indicate that the bladder cells of HIC patients are significantly more disrupted than those of NHIC patients, with increased protein misfolding and decreased cellular adhesion leading to an inflammatory response and painful lesions in the bladder wall which heal slowly. These results are important, as they may lead to a greater understanding of the underlying causes of IC, as well as potential new therapeutic targets for HIC or NHIC.

## Data Availability

The datasets generated and analyzed during the current study are available from the corresponding author on reasonable request.

## Abbreviations

D: Disease
IC/BPS: Interstitial cystitis/bladder pain syndrome
HIC: Hunner’s interstitial cystitis
NHIC: Non-Hunner’s interstitial cystitis
nHPLC-MS/MS: Nano high performance liquid chromatography linked to tandem mass spectrometry
NDA: Non-disease apparent
ICSI: Interstitial Cystitis Symptom Index
ICPI: Interstitial Cystitis Problem Index
MS: Mass spectrometry
MS/MS: Tandem mass spectrometry
NSAF: Normalized spectral abundance factor
ER: Endoplasmic reticulum
HLA: Human leukocyte antigen
